# Treating sleep-disordered breathing of idiopathic pulmonary fibrosis patients with CPAP and nocturnal oxygen treatment. A pilot study: Sleep-disordered breathing treatment in IPF

**DOI:** 10.1186/s12931-024-02871-6

**Published:** 2024-06-18

**Authors:** Jaume Bordas-Martinez, Neus Salord, Vanesa Vicens-Zygmunt, João Carmezim, Sandra Pérez, Eliseo Prado, María Calvo, Rosana Blavia, Guadalupe Bermudo, Salud Santos, Carmen Monasterio, María Molina-Molina

**Affiliations:** 1 Interstitial Lung Disease Unit, Respiratory Department, Bellvitge University Hospital. IDIBELL. CIBERES, University of Barcelona. -Hospitalet de Llobregat (Barcelona), Barcelona, Spain; 2 Sleep Unit, Respiratory Department, Bellvitge University Hospital, IDIBELL. University of Barcelona. - Hospitalet de Llobregat (Barcelona), 08907 Barcelona, Spain; 3 Respiratory Department, Granollers University Hospital. -Granollers (Barcelona), Barcelona, Spain; 4 Biostatistics Unit, IDIBELL. Hospitalet de Llobregat (Barcelona), Barcelona, Spain; 5 Respiratory Department, Hospital Moises Broggi. -Sant Joan Despí, Barcelona, Spain

**Keywords:** Idiopathic pulmonary fibrosis, Sleep, Apnoea, Hypoxemia, Biomarkers, CPAP, Supplemental oxygen therapy

## Abstract

**Introduction:**

Sleep-disordered breathing (SDB) is a major comorbidity in idiopathic pulmonary fibrosis (IPF) and is associated with a poor outcome. There is a lack of knowledge regarding the impact of SDB treatment on IPF. We assessed at one year: (1) the effect of CPAP and/or nocturnal oxygen therapy on IPF regarding lung function, blood mediators, and quality of life; (2) adherence to SDB treatment and SDB changes.

**Methodology:**

This is a prospective study of consecutive newly diagnosed IPF patients initiating anti-fibrotic treatment. Lung function, polysomnography, blood tests and quality of life questionnaires were performed at inclusion and after one year. Patients were classified as obstructive sleep apnoea (OSA), central sleep apnoea (CSA), and sleep-sustained hypoxemia (SSH). SDB therapy (CPAP and/or nocturnal oxygen therapy) was initiated if needed.

**Results:**

Fifty patients were enrolled (36% had OSA, 22% CSA, and 12% SSH). CPAP was started in 54% of patients and nocturnal oxygen therapy in 16%. At one-year, polysomnography found improved parameters, though 17% of patients had to add nocturnal oxygen therapy or CPAP, while 33% presented SDB onset at this second polysomnography. CPAP compliance at one year was 6.74 h/night (SD 0.74). After one year, matrix metalloproteinase-1 decreased in OSA and CSA (*p* = 0.029; *p* = 0.027), C-reactive protein in OSA (*p* = 0.045), and surfactant protein D in CSA group (*p* = 0.074). There was no significant change in lung function.

**Conclusions:**

Treatment of SBD with CPAP and NOT can be well tolerated with a high compliance. IPF patients may exhibit SDB progression and require periodic re-assessment. Further studies to evaluate the impact of SDB treatment on lung function and serological mediators are needed.

**Supplementary Information:**

The online version contains supplementary material available at 10.1186/s12931-024-02871-6.

## Introduction

Idiopathic pulmonary fibrosis (IPF) is a fatal progressive fibrotic interstitial lung disease (ILD) [1–3]. Antifibrotic drugs slow the decline of lung function [4] and improve prognosis, which remains poor [5]. Sleep-disordered breathing (SDB) is a highly prevalent IPF comorbidity, ranging from 45 to 90% depending on the methodology used [6–11]. SDB involves different entities, and while obstructive sleep apnoea (OSA) and nocturnal hypoxemia have been associated with poor outcomes in IPF [6, 12, 13], the impact of central sleep apnoea (CSA) remains poorly understood [14, 15].

OSA and intermittent hypoxemia have been associated with ILD and an increment in the blood mediators of pulmonary interstitial remodelling and alveolar epithelial damage [14, 16–18]. The progression of pulmonary fibrosis has been associated with the development of SDB through a mechanism that is still under debate [14, 19–21].

The treatment of SDB in IPF patients seems to improve quality of life and prognosis despite the scarce number of studies [11, 22, 23]. Some methodological limitations include the absence of a generalized use of antifibrotic drug therapy [4, 5] and self-selection bias owing to a high refusal rate and poor compliance with continuous positive airway pressure (CPAP) [24]. There is limited data regarding the effect of using nocturnal oxygen therapy (NOT) in ILD patients and, to the best of our knowledge, there is no data regarding the effect of treating CSA in patients with ILD [25–27].

Therefore, this study of IPF patients aimed to assess: (1) the impact of personalized SDB treatment by CPAP and/or nocturnal oxygen therapy on lung function, blood mediators and quality of life; (2) tolerance of and adherence to SBD treatment and sleep-disordered breathing changes after one year of treatment and follow-up.

## Methodology

This is a prospective pilot study of consecutive newly diagnosed IPF patients who were systematically studied for SBD and treated with CPAP and/or NOT depending on the type of SDB. Inclusion criteria was a confident IPF diagnosis by a multidisciplinary committee [2, 3]. Exclusion criteria included other life-threatening or unstable diseases.

Polysomnography (PSG), pulmonary function test, 6-minute walking test (6MWT), blood test, and questionnaires were performed at baseline and after one year. SDBs were classified as previously described [7]: (A) OSA when the apnoea-hypopnea index (AHI) was ≥ 15/h and obstructive events were predominant (≥ 50%); (B) CSA when AHI ≥ 15/h and there were predominant central events; (C) sleep sustained hypoxemia (SSH) when total sleep time under SpO2 88% (TST88) > 5 min and AHI < 15/h; (D) No-SDB when AHI < 15/h and TST88 ≤ 5 min. PSG at one year was performed under treatment if the patients was receiving it or basally if no treatment had been started.

Blood mediators included C-reactive protein (CRP), lactate dehydrogenase (LDH), N-terminal pro-B-type natriuretic peptide, interleukin 6 (IL-6), matrix metalloproteinase-1 (MMP-1), MMP-7, MMP-9, surfactant protein D (SP-D), tenascin-c large, Krebs von den Lungen 6 (KL-6), advanced glycation end-products (AGEs), and receptor of AGEs (RAGEs). Sleep quality and sleep habits questionnaires, the Epworth Sleepiness Scale (ESS), the Functional Outcomes of Sleep Questionnaire short version, EuroQol 5D-5 L, and the King’s Brief Interstitial Lung Disease questionnaire were analysed. Comorbidities were assessed through the Beck Depression Inventory-II, the 7-item Generalized Anxiety Disorder Scale and the Gastroesophageal Reflux Disease Questionnaire. All patients received anti-fibrotic medication (nintedanib or pirfenidone) after the diagnosis and at least two weeks before the first blood sample was obtained.

**Fig. 1:**
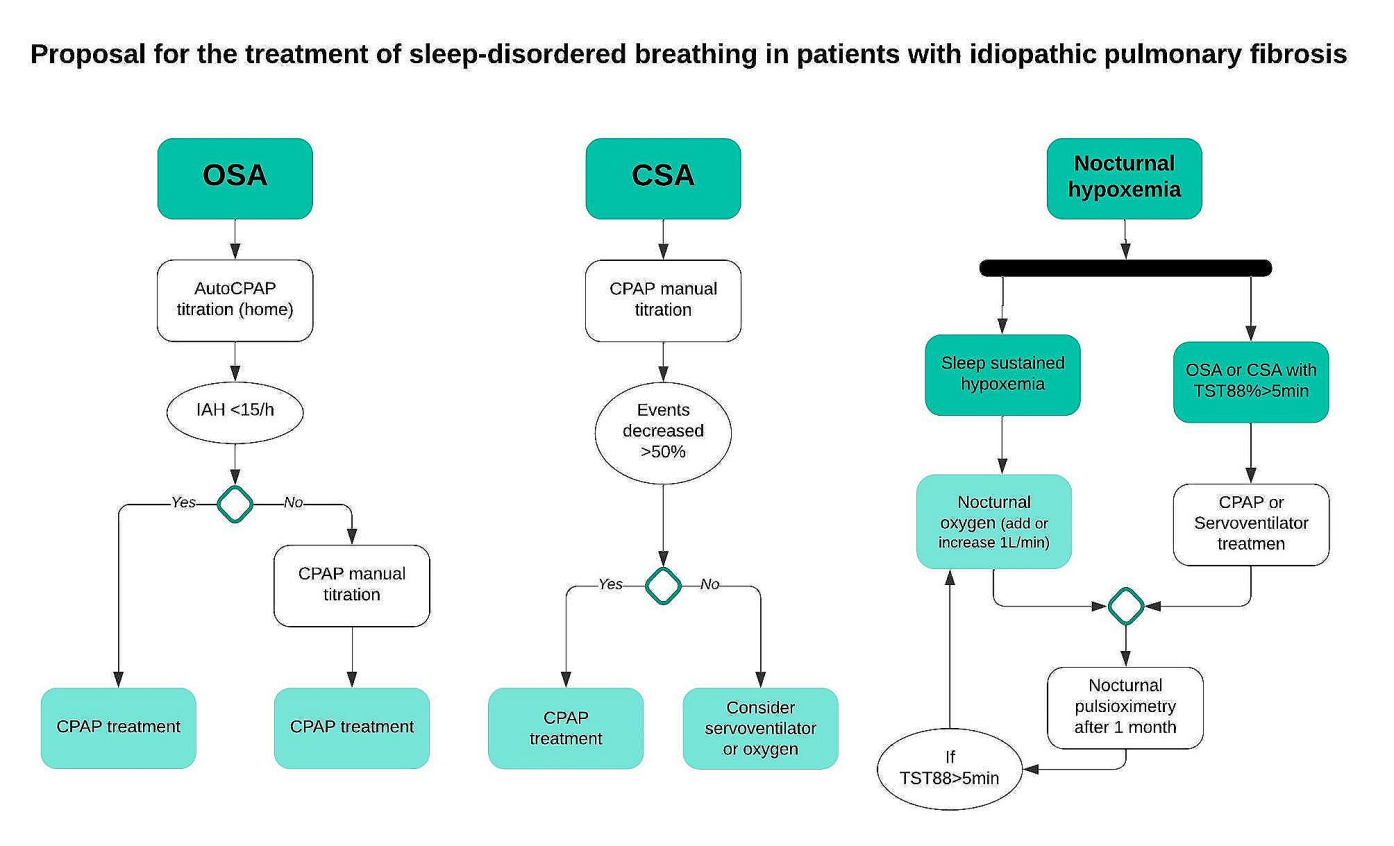
Proposal for the treatment of sleep-disordered breathing in patients with idiopathic pulmonary fibrosis. OSA: obstructive sleep apnoea; CSA: central sleep apnoea; AHI: apnoea-hypopnea index; TST88: total sleep time under SpO2 88%; CPAP: continuous positive airway pressure; O2: supplemental oxygen

The SDB treatment decision was based on the particular SDB (Fig. 1). Patients with moderate or severe OSA were treated with CPAP after a home autoCPAP titration [28, 29]. For those with moderate or severe CSA the treatment was individualized: they underwent manual CPAP titration and started CPAP if events decreased ≥ 50% [30]; if events did not decrease > 50%, servoventilation or oxygen therapy were considered and decided in each individualized case depending on IAH severity and the degree of nocturnal hypoxemia. Patients with nocturnal hypoxia using the criterion TST88 ≥ 5 min, started nocturnal oxygen therapy (NOT) [31, 32]. After one month of SDB treatment, patients with TST88 ≥ 5 min were re-assessed by nocturnal pulse-oximetry. Clinical follow-up following the standard of care assessment for IPF treatment and the SDB treatment by specialist nurses was performed at one, three, six and 12 months.

### Statistics

Continuous variables were presented with mean and standard deviation, except for those with asymmetry or lack of normality, which were described with median and interquartile range. Categorical variables were reported as the number of cases and the percentage of the total. Paired t-tests were used for normally distributed variables, the Wilcoxon signed-rank test for non-normally distributed variables and the McNemar test for categorical variables. Using logistic regression models, the crude effect and the effect adjusted by forced vital capacity (FVC), diffusing capacity for carbon monoxide (DLCO) and age were analysed. Statistical analyses were performed with R software version 4.2.1 or higher and STATA 17.

## Results

### Study population

Fifty patients were enrolled (Table 1) and 72% were male. Mean age was 72.6 years and only 28% of cases were obese (BMI > 30 kg/m2). The sleep study identified 70% of patients as having some type of SDB encompassing 36% OSA, 22% CSA and 12% SSH. The baseline characteristics have been previously reported in more detail [14]. Importantly, CSA cases were detected by coding non-obstructive hypopneas and these patients presented a predominance of hypopneas, compared to apnoeas, and did not present periodic breathing. CPAP was started in all OSA patients and 82% of CSA patients (nine patients), while NOT was initiated in SSH patients and 18% of CSA patients (two patients) (Table 2). Two patients died during follow-up (one OSA and one CSA).

**Table 1:** **Characteristics of the study population at baseline**

	Prospective Cohort
	*N* = 50
Age, Mean (SD)	72.6 (7.35)
Sex (male),	36 (72.0%)
**Smoking Exposure, N (%)**:	
Never	18 (36.0%)
Former smoker	32 (64.0%)
Arterial Hypertension, N (%)	32 (64.0%)
Dyslipidemia, N (%)	24 (48.0%)
Diabetes Mellitus, N (%)	16 (32.0%)
Heart Disease, N (%)	11 (22.0%)
Echocardiography Pulmonary Hypertension, N (%)	20 (40.0%)
Chronic Kidney Failure, N (%)	5 (10.0%)
GERD, N (%)	23 (46.0%)
Depression-Anxiety, N (%)	13 (26.0%)
Malignant Disease, N (%)	8 (16.0%)
Obesity (BMI ≥ 30)	14 (28.0%)
**Thorax HRCT Pattern, N (%)**:	
Definite UIP pattern	23 (46.0%)
Probable UIP pattern	17 (34.0%)
Pattern indeterminate for UIP	10 (20.0%)
Emphysema (extension < 15%), N (%)	10 (20.0%)
**Biopsy, N (%)**:	
Cryobiopsy	3 (6.00%)
Surgical biopsy	9 (18.0%)
**Anathomopathological Patterns, N (%)**:	
NIU	9 (75.0%)
Probable NIU	3 (25.0%)
**Pulmonary Lung function**, Median [Q1;Q3]	
BMI	27.0 [26.0;30.0]
FVC (%)	94.0 [81.5;104]
FVC (mL)	2615 [2085;3168]
FEV1/FVC	82.7 [78.2;86.4]
TLC (%)	77.0 [67.2;84.5]
TLC (mL)	4345 [3228;5040]
DLCO (%)	55.0 [46.5;70.0]
KCO (%)	82.0 [70.0;94.8]
6MWD distance (m)	425 [365;462]
6MWD minimal SpO2%	91.0 [87.0;94.0]
**Antifibrotic Treatment, N (%)**:	
Pirfenidone	13 (26.0%)
Nintedanib	37 (74.0%)

SD: Standard deviation; COPD: Chronic obstructive pulmonary disease; GERD: gastroesophageal reflux disease; CPFE: combined pulmonary fibrosis and emphysema; BMI: body mass index; HRC: High-Resolution Computed Tomography; FVC: forced vital capacity; DLCO: diffusing capacity of the lungs for carbon monoxide

**Table 2:** Sleep-disordered breathing treatment

SDBs treatment		OSA	CSA	SSH	No-SDB
		*N* = 18	*N* = 11	*N* = 6	*N* = 15
Baseline	No treatment				15 (100%)
	NOT		2 (18%)	6 (100%)	
	CPAP	18 (100%)	9 (82%)		
	CPAP + NOT				
After 1-year	No treatment				10 (67%)
	NOT		2 (18%)	5 (83%)	2 (13%)
	CPAP	16 (89%)	6 (55%)		3 (20%)
	CPAP + NOT	2 (11%)	3 (27%)	1 (17%)	

OSA: obstructive sleep apnea; CSA: central sleep apnea; SSH: sleep sustained hypoxemia; SBD: sleep-disordered breathing; CPAP: continuous positive airway pressure; NOT: nocturnal oxygen therapy

#### Changes in lung function, blood mediators and QoL

**Fig. 2:**
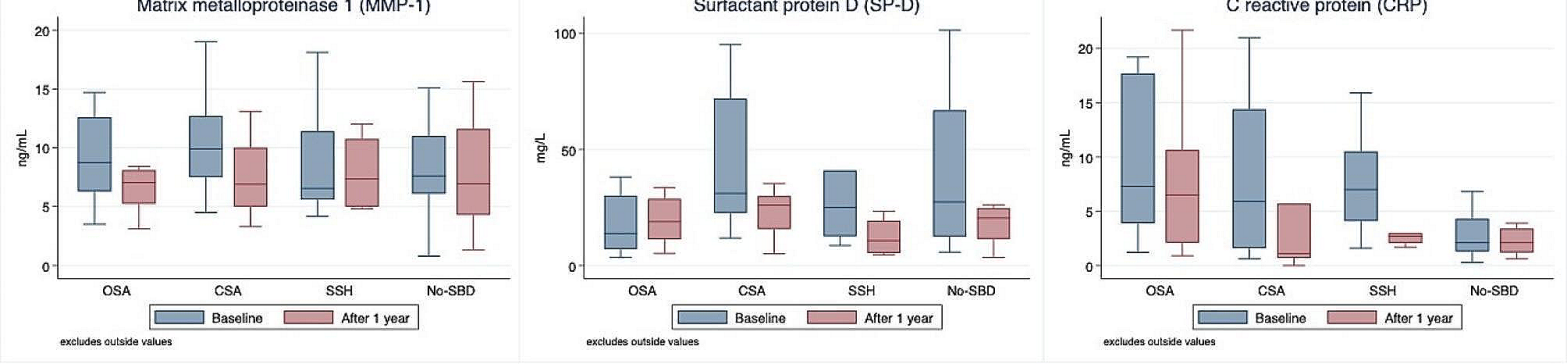
Blood biological mediators at baseline and at one year by type of SBD. Outsider values were excluded. OSA: obstructive sleep apnoea; CSA: central sleep apnoea; SSH: sleep-sustained hypoxemia; No- SDB: No sleep-disordered breathing; MMP-1: matrix metalloproteinase 1; SP-D: surfactant protein D ; CRP: C-reactive protein

Data are summarized in Table 3 according to SBD classification. At baseline, the mean FVC was lower in the SSH group (below 80%). After one year, lung function did not significantly change. Regarding the higher serum concentration of MMP-1 at baseline (Fig. 2) in the OSA and CSA groups, a significant decrease was observed after one year (*p* = 0.029 and *p* = 0.027, respectively). Furthermore, patients with OSA also showed a significant decrease of CRP in blood samples (*p* = 0.045) and the CSA group showed a trend to reduction of SP-D (*p* = 0.07). No significant differences were found when comparing the 1-year changes in lung function and blood mediators with the non-SBD group. The questionnaires (Supplemental Table 1) showed a significant improvement in ESS in OSA patients, with no other relevant changes.

**Table 3:** SDB groups. All data are presented as Median [Q1;Q3]

				OSA (*N* 1)							CSA (*N* 9)				SSH (*N* 4)				No-SDB (*N* 14)	
		Baseline		1-year		*p*.value			Baseline	1-year		*p*.value		Baseline	1-year	*p*.value		Baseline	1-year	*p*.value
Lung function test																				
BMI	28.5 [27;33.43]		29.29 [27.42;33.43]		0.464				26 [25;30.06]	25.82 [23.5;30.06]		0.250		28.5 [26.75;28.4]	26.48 [24.6;28.4]	0.125		27 [25.45;28.47]	26.53 [25.6;28.47]	0.391
FVC (mL)	2800 [2370;3167.5]		2780 [2172.5;3167.5]		0.132				2790 [2640;3440]	2810 [2480;3440]		0.652		2060 [1997.5;2427.5]	2020 [1910;2427.5]	1.000		2310 [1907.5;3290]	2360 [1790;3290]	0.432
FVC (%)	92.5 [86.75;113.75]		90 [83.5;113.75]		0.363				101 [83;117]	108 [78;117]		0.343		72 [62.25;84]	71.5 [61.25;84]	0.250		97 [93;105.75]	99 [85.25;105.75]	0.615
FEV1/FVC	81.98 [78.26;84.33]		80.31 [78.1;84.33]		0.632				82.62 [74.46;83.9]	78.52 [69.7;83.9]		0.027		80.91 [79.05;88.99]	83.21 [79.09;88.99]	1.000		82.75 [78.74;88.34]	84.75 [76.95;88.34]	0.903
TLC (mL)	4430 [3960;4640]		4180 [3700;4640]		0.083				4790 [4390;5730]	4820 [4150;5730]		0.359		3310 [3250;3660]	3470 [3250;3660]	0.773		4240 [2930;4730]	4080 [2960;4730]	0.162
TLC (%)	77 [73.5;83]		74 [71;83]		0.123				77 [71;91]	86 [67;91]		0.301		62 [60;70]	67 [64;70]	1.000		80 [71;80]	73 [70;80]	0.233
DLCO (%)	56.5 [53.25;70.25]		56 [49.5;70.25]		0.029				51 [46;77]	58 [42;77]		0.552		71 [60;65]	57 [49;65]	0.371		62.5 [49.5;60.25]	55 [45.75;60.25]	0.012
KCO (%)	82.5 [69.75;103.25]		89 [72;103.25]		0.518				81 [65;103]	77 [71;103]		0.905		97 [95.5;114.5]	99 [93;114.5]	1.000		84 [79.5;86]	79.5 [77.75;86]	0.024
6MWD distance (m)	431.5 [391;492]		437 [377;492]		1.000				433 [423;447]	423 [384;447]		0.734		374.5 [341;382.5]	329 [293;382.5]	0.789		435 [398.25;446.25]	416.5 [355.75;446.25]	0.069
6MWD minimal SpO2%	90.5 [87.75;95]		90.5 [88.75;95]		0.861				92 [90;96]	93 [91;96]		0.325		90.5 [88;90.5]	90 [90;90.5]	0.875		92 [89.75;93.75]	90.5 [87;93.75]	0.423
Blood biological mediators																				
CRP	7.3 [3.5;10.62]		6.5 [2.2;10.62]		0.045				2.2 [1.6;5.7]	1.1 [0.7;5.7]		0.496		6.05 [3.47;3]	2.7 [2.22;3]	0.250		2.1 [1.35;3.28]	2.1 [1.22;3.28]	0.485
LDH	200.5 [170.25;211.25]		194.5 [169.75;211.25]		0.856				203 [190;214]	204 [177;214]		0.129		166 [158.5;167]	154 [153;167]	1.000		200 [179.75;233.5]	193 [173.5;233.5]	0.576
NT-BNP	83 [25.5;214.5]		187 [169.5;214.5]		0.229				214 [135;214]	204 [177;214]		0.734		32 [25.5;167]	154 [153;167]	0.250		81 [31;236]	198 [175;236]	0.002
IL-6	11.52 [7.18;27.52]		17.62 [11;27.52]		0.029				9.85 [8.83;36.74]	12.56 [11.34;36.74]		0.02		11.74 [10.45;15.56]	13.87 [11.66;15.56]	0.875		8.59 [6.5;17.11]	9.49 [7.22;17.11]	0.042
MMP-1	8.75 [6.12;7.92]		7.02 [5.27;7.92]		0.029				10.2 [7.5;9.98]	6.91 [5.01;9.98]		0.027		5.6 [5.25;10.11]	7.31 [5.05;10.11]	1.000		7.5 [6.12;11.5]	6.98 [4.59;11.5]	0.391
MMP-7	5.54 [4.35;7.37]		5.71 [4.65;7.37]		0.495				7.73 [6.74;7.98]	6.83 [5.94;7.98]		0.57		6.25 [5.73;8.22]	6.84 [6.18;8.22]	0.875		5.4 [5.17;7.28]	5.77 [4.75;7.28]	0.67
MMP-9	240.06 [186.99;591.38]		487.24 [413.61;591.38]		< 0.001				348.2 [293.95;776.08]	640.07 [605.72;776.08]		0.004		246.55 [206.21;590.34]	411.6 [268.01;590.34]	0.625		161.82 [125.18;439.26]	306.89 [228.73;439.26]	< 0.001
FNIII-C	92.93 [73.01;184.53]		151.48 [131.58;184.53]		< 0.001				103.67 [85.76;261.14]	193.72 [109.59;261.14]		0.055		96.58 [89.8;191.3]	153.66 [131.12;191.3]	0.250		97.27 [69.48;194.79]	174.92 [125.97;194.79]	< 0.001
AGEs/RAGEs	15.03 [9.64;20.88]		12.07 [8.92;20.88]		0.86				17.63 [15.82;18.54]	10.91 [6.58;18.54]		0.027		15.4 [9.87;21.59]	14.39 [8.46;21.59]	0.625		18.11 [15.69;16.95]	12.31 [7.33;16.95]	0.02
SP-D	12.67 [7.11;28.13]		18.88 [12.16;28.13]		0.562				43.4 [29.16;29.93]	25.97 [15.77;29.93]		0.074		25.06 [19.37;17.36]	10.68 [5.68;17.36]	0.250		22.34 [12.65;24.21]	20.46 [11.65;24.21]	0.626
KL-6	804.5 [608.5;1242.25]		882 [532.25;1242.25]		0.782				856 [512;991]	855 [599;991]		0.213		410.5 [388.5;841.75]	433 [390.25;841.75]	0.375		797 [629.25;1227.75]	897 [646.25;1227.75]	0.626
Sleep study																				
SE (%)	71.75 [62.62;80.45]		67.1 [61.7;80.45]		0.453				74.1 [55.4;75]	69.8 [51.1;75]		0.91		67.8 [56.35;73.22]	65.55 [55.9;73.22]	1		78.1 [62.7;86.35]	72.2 [64.6;86.35]	0.577
N1 (% TST)	19.4 [15.2;14.95]		12.95 [8.22;14.95]		0.016				13.5 [4.9;22.8]	16.1 [10.8;22.8]		0.359		11.3 [8.57;19]	13.5 [9.85;19]	0.375		9.3 [6.85;15.8]	14.1 [5.5;15.8]	0.444
N2 (% TST)	39.4 [34.95;45.07]		38.25 [28.9;45.07]		0.669				36.9 [27.5;41.1]	31.7 [31.4;41.1]		0.91		32.6 [29.42;38]	35.6 [31.83;38]	0.625		38.4 [33.95;43.35]	38.7 [35.75;43.35]	0.45
N3 (% TST)	25.9 [16.55;38.03]		28.15 [25.2;38.03]		0.187				38.4 [32;31.3]	26.6 [24.1;31.3]		0.074		38.7 [32.6;38.05]	30.55 [26.32;38.05]	0.25		34.6 [21.4;36.7]	33.6 [23.35;36.7]	0.919
REM (% TST)	14.4 [6.7;21.88]		16.5 [10.47;21.88]		0.495				17.6 [9.3;20.3]	20.1 [12.8;20.3]		1		20.15 [15.82;18.22]	15.9 [14.3;18.22]	1		17.4 [12.05;21.55]	17.4 [12.65;21.55]	0.278
AI (events/h)	3.65 [1.57;0.35]		0 [0;0.35]		< 0.001				10.27 [1.19;0.7]	0 [0;0.7]		0.021		0 [0;1.35]	0.5 [0;1.35]	0.423		0.63 [0;1.05]	0.55 [0;1.05]	0.919
HI (events/h)	20.47 [17.63;8.9]		4.2 [1.53;8.9]		0.002				24.46 [19.91;10.1]	9.5 [4.1;10.1]		0.008		11.36 [10.01;19.22]	10.25 [3.17;19.22]	0.875		6.37 [3.36;11.07]	7.6 [4.62;11.07]	0.091
AHI (events/h)	25.5 [21.23;9.28]		4.2 [1.65;9.28]		< 0.001				30.4 [22.8;10.1]	10.1 [4.8;10.1]		0.004		11.45 [10.18;20.6]	10.75 [3.17;20.6]	1		7.2 [4.9;12.2]	9.5 [5.3;12.2]	0.146
Mean SpO2 (%)	94 [93;96]		95 [93.75;96]		0.008				93 [92;97]	95 [94;97]		0.121		92.5 [91.75;96.5]	95 [94;96.5]	0.098		95 [94;95.75]	94.5 [93.25;95.75]	0.198
Lowest SpO2 (%)	81 [78.75;91]		89 [88;91]		0.015				82 [80;89]	86 [85;89]		0.092		83.5 [82.75;87.75]	86.5 [86;87.75]	0.125		89 [88;88.75]	86 [83.25;88.75]	0.005
ODI (events/h)	19.55 [16.68;10.12]		5 [2.42;10.12]		< 0.001				48.3 [23.2;11.5]	7.4 [1.7;11.5]		0.004		12.1 [9.58;12.57]	6.4 [0.45;12.57]	0.125		4.6 [2.42;9.53]	5.2 [3.62;9.53]	0.124
TST90 (%)	1.9 [1;0.45]		0.1 [0;0.45]		0.012				12.4 [4.7;2]	0.1 [0;2]		0.004		7.05 [6.33;0.8]	0.4 [0;0.8]	0.125		0.1 [0;1.65]	0.1 [0;1.65]	0.148
TST88 (%)	0.6 [0.18;0]		0 [0;0]		0.01				3.1 [0.8;0.2]	0 [0;0.2]		0.004		1.8 [1.8;0.12]	0.05 [0;0.12]	0.125		0 [0;0.2]	0.05 [0;0.2]	0.05
TST88 (minutes)	2.65 [0.75;0.03]		0 [0;0.03]		0.007				14.3 [5.2;1]	0.1 [0;1]		0.004		8.3 [7.85;0.62]	0.3 [0;0.62]	0.125		0 [0;0.95]	0.25 [0;0.95]	0.041
PLM index	2.9 [0;41.88]		8.8 [0;41.88]		0.059				0 [0;3.4]	1 [0;3.4]		0.281		0 [0;1.35]	0 [0;1.35]	1		0.8 [0;14.55]	6.5 [0;14.55]	0.042

All data are presented as Median [Q1; Q3]. OSA: obstructive sleep apnea; CSA: central sleep apnea; SSH: sleep sustained hypoxemia; SBD: sleep-disordered breathing; BMI: body mass index; FVC: forced vital capacity; FEV1: forced expiratory volume in 1 s; TLC: total lung capacity; DLCO: diffusing capacity of the lungs for carbon monoxide; KCO: carbon monoxide transfer coefficient; 6MWT: 6-min walking test; SpO2: arterial oxygen saturation measured by pulse oximetry; CRP: [1]; LDH: lactate dehydrogenase; NT-BNP: N-terminal pro-B-type natriuretic peptide; IL-6: human Interleukin 6; MMP-1: matrix metalloproteinase 1; MMP-7: matrix metalloproteinase 7; MMP-9: matrix metalloproteinase 9; FNIII-C: tenascin-c large ; AGEs: advanced glycation end-products; RAGEs: receptor for advanced glycation end products; SP-D: surfactant protein D; KL-6: Krebs von den Lungen-6; SE: Sleep Efficiency; REM: rapid eye movement; AI: Apnea index; HI: hypopnea index; AHI: apnea–hypopnea index; SpO2: arterial oxygen saturation measured by pulse oximetry; ODI: oxygen desaturation index; TST90: percentage of TST with SpO2 &lt; 90%; TST88: percentage of TST with SpO2 &lt; 88%; PML: Periodic movement legs,

#### Polysomnographic changes

The PSG study under SDB treatment at 1-year compared to the baseline showed a significant decrease in AHI and TST90 in the OSA and CSA groups, and a non-significant increase in mean oxygen saturation in the SHH group. The No-SDB group showed a significant decrease only in lowest oxygen saturation in basal PSG at one year. Concerning sleep phases, only the OSA group had a significant decrease in N1 (% TST).

The PSG study under SBD treatment at one year also revealed the need to add NOT to CPAP in two out of 16 OSA group patients and three out of nine patients in the CSA group (Table 2). CPAP was added to one patient in the SSH group initially treated with NOT. In the non-SDB group, the sleep study was repeated without treatment and it disclosed the existence of SDB in five out of 15 patients, requiring starting CPAP in three and NOT in two patients. The changes at one year in FVC, DLCO and weight in this group were not predictive of SDB progression in a logistic model.

### SDB treatment compliance, side effects and patient satisfaction

CPAP compliance at one year was 6.74 h/night (SD 0.74) and all patients had a compliance higher than 5 h/night. Mean CPAP pressure was 7 mmHg [IQR 6;9] (OSA group: 7 mmHg [IQR 6;8] and CSA group: 8 mmHg [IQR 6;9]). The main reported adverse effect (Supplemental Table 2) was dry mouth, but no treatment discontinuation was associated with side effects. Regarding the Patient-Reported Experience Measures (PREMs) (Supplemental Table 3), satisfaction with the sleep study and SDB management was nearly 100%.

## Discussion

This pilot study highlights the fact that the personalized management and treatment of SDBs with CPAP and NOT in patients with IPF is feasible, resulting in high CPAP compliance, few side effects and a high level of satisfaction. Most patients with SDBs do not present typical symptoms and a SDB may appear at any time during the follow-up.

There is very limited evidence regarding the treatment of SBD in IPF patients, evidence for OSA is scarce [33]; it was anecdotal for nocturnal hypoxia in ILD [25–27], and absent for CSA. Therefore, our proposal was a proof of concept treating each condition individually according to the recommendations of the respective guidelines, and applying a personalized approach in order to achieve the optimal correction of each sleep disorder. Therefore, OSA was treated with CPAP with good results. As the treatment of CSA is not established for patients with FPI [30], as a general rule, we treated it with CPAP if manual CPAP titration decreased events ≥ 50%. This objective was not achieved in only two patients who had low basal IAH (16.0/h and 21.1/h) and a TST88 ≥ 5 min and who received nocturnal oxygen, achieving a good correction in control nocturnal oximetry. For this reason, no patient was submitted to serventilation. Patients with SSH were treated with NOT [32, 34]. This approach, and the cut-off points used to initiate each treatment, should be validated in future multicentre studies and larger real-world settings.

There were no significant differences in lung function after one year. Other studies treating patients with IPF and OSA by CPAP did not measure pulmonary function, but an improvement in survival was reported [11, 22]. However, a longer follow-up of larger a larger cohort and a control group would be required to better assess the potential impact of SDB treatment on IPF outcomes.

Increased levels of oxidative, inflammatory and profibrotic serological mediators were observed in IPF patients with SDB in previous studies [10, 14, 16–18]. Our previous study characterized different SDBs in IPF patients and their relationship with lung function and blood mediators [14]. We hypothesized that different SDBs may be associated with distinct metabolic pathways. Apnoea groups presented higher levels of MMP-1 at baseline, which decreased significantly after one year of SDB treatment. MMP-1 is a profibrotic metalloproteinase related to hypoxia-inducible factor 1 (HIF-1) [35], which is increased in intermittent hypoxia, but not in sustained hypoxia [36]. Furthermore, OSA and CSA present certain differences. As for non-ILD OSA patients [37], CRP was reduced after one year in the OSA group. On the other hand, SP-D was reduced to a figure close to statistical significance (*p* = 0.07) in CSA patients. SP-D is a blood mediator of epithelial alveolar tissue [38] associated with an increased death risk rate in IPF patients [39]. SP-D may be up and down-regulated by HIF-1 in acute and persistent hypoxia, respectively [40], and higher levels of hypopnea index were associated with a decrease in SP-D blood levels [41]. However, it is important to note that our study has an exploratory character and these changes cannot be extended to the treatment of SDB because many confounding factors, such as the initiation of antifibrotic treatment, may lead to erroneous conclusions. Future prospective randomized studies with longer follow-ups should evaluate these findings.

On the other hand, there is mounting evidence that non-physiological mechanical lung strain can produce an activation of pro-inflammatory and pro-fibrotic pathways that results in the progression of pulmonary fibrosis [42, 43]. Therefore, the long-term safety of CPAP treatment depending on the required positive end expiratory pressure (PEEP) levels should be considered in the future. Our data showed no significant changes in lung function. In addition, apnoea and hypopnea events in IPF patients have been also suggested as a pro-fibrotic mechanism [21, 44, 45]. CPAP treatment could reduce repetitive lung strain and hypoxia-related damage. The accurate estimation of low CPAP pressure required may also have been a key factor in preventing mechanical damage and reducing the harmful effect of SDBs [43].

The high CPAP acceptance and compliance shown by the patients in our study contrasts with previous studies with IPF patients. In two similar studies, 8% and 16% of IPF candidate patients withdrew from CPAP treatment within the first month [11, 23]. In line with our data, a recent study did not report any CPAP refusal or discontinuation [22]. Regarding CPAP compliance, 33% and 62% reported poor adherence (≥ 4 h/night) [11, 22]. Conversely, none of the patients in our series, had an adherence of less than five hours per night. The reasons for this good SDB treatment compliance are unknown, as they were not the focus of this study. However, a combination of comprehensive information on SDB, personalized SDB treatment and a close follow-up with an assessment of side effects may have had an influence.

One-third of patients without baseline SDB had some SDB at one year of control and had to start treatment, while 17% of patients treated for SBD had to increase their SDB treatment only one year later. Surprisingly, that was not associated with lung function decline or weight change. If this finding is confirmed in further series, sleep studies should be considered not only at diagnosis [8–10, 46, 47], but also during follow-up.

There were no significant differences in quality of life at one year. The COVID-19 outbreak may have negatively impacted quality of life results, which may explain the differences with regard to previous studies [11, 22, 23]. However, the satisfaction score and PREMs of sleep screening, treatment and follow-up were high, suggesting that the process does not cause great inconvenience to patients.

### Strengths and limitations

Limited sample size is the main limitation of this exploratory pilot study and constrained the statistical analyses, particularly when examining distinct SDB types such as the SSH group. Furthermore, although the enrolment of all patients after initiating antifibrotic drugs better enabled the assessment of SDB outcomes, its effect on respiratory function and serum mediators could not be completely attributed to SDB treatment and the results should be considered with caution before being properly validated. All patients were treated with antifibrotics, whereas previous studies did not report or reported only partially on the use of antifibrotic drug treatment [11, 22, 23]. The holistic diagnosis and management of SDB is another remarkable strength of the study, since it does not preclude different SDBs such as CSA [22], or treating nocturnal hypoxia with NOT and not only OSA by CPAP [11, 22, 23]. Adherence may impact SDB outcomes, but due to the high level of compliance among the included patients, we were not able to analyse outcomes depending on the level of adherence. Finally, these results must be interpreted with caution, not only due to the limited number of patients, but also other potential confounding factors such as the absence of a control group with SDB but without treatment.

## Conclusions

Our study suggests that a personalized selection of SDB treatment including CPAP and/or NOT involving a comprehensive follow-up of the therapy may be well-accepted and achieve high compliance. SDB may appear or increase SDB treatment requirements only one year after their study. Therefore, not only sleep studies in IPF patients should be considered, but also periodic re-assessment. Future randomized and longer follow-up studies are needed to evaluate the impact of SDB treatment on both lung function and biological mediators.

### Electronic supplementary material

Below is the link to the electronic supplementary material.

Supplementary Material 1

## Data Availability

The data that support the findings of this study are available from the corresponding author upon reasonable request.

## Abbreviations

IPF: Idiopathic pulmonary fibrosis
ILD: Interstitial lung diseases
SDB: Sleep-disordered breathing
OSA: Obstructive sleep apnoea
CSA: Central sleep apnoea
CPAP: Continuous positive airway pressure
NOT: Nocturnal oxygen therapy
6MWT: 6-minute walking tests
PSG: Polysomnography
SSH: Sleep-sustained hypoxemia
AHI: Apnoea-hypopnea index
TST: Total sleep time
TST90/88: Total sleep time under SpO2 90% /88%
CRP: C-reactive protein
LDH: Lactate dehydrogenase
NT-BNP: N-terminal pro-B-type natriuretic peptide
IL-6: Interleukin 6
MMP: Matrix metalloproteinase
SP-D: Surfactant protein D
FNIII-C: Tenascin-c large
KL-6: Krebs von den Lungen 6
AGEs: Advanced glycation end-products
RAGEs: Receptor of advanced glycation end-products
ESS: Epworth sleepiness scale
FOSQ-10: Functional outcomes of sleep questionnaire short version
EQ-5D-5L: EuroQol 5D-5 L
K-BILD: King’s brief interstitial lung disease questionnaire
BDI-II: Beck depression inventory-II
GAD-7: 7-item generalized anxiety disorder scale
GerdQ: Gastroesophageal reflux disease questionnaire
FVC: Forced vital capacity
DLCO: Diffusing capacity of the lungs for carbon monoxide
BMI: Body mass index
GERD: Gastroesophageal reflux disease
SD: Standard deviation
PREMs: Patient-Reported Experience Measures
IQR: Interquartile range
PEEP: Positive end expiratory pressure
