# Supplementary material for: Treating sleep-disordered breathing of idiopathic pulmonary fibrosis patients with CPAP and nocturnal oxygen treatment. A pilot study: Sleep-disordered breathing treatment in IPF
Source: Respir Res. 2024 Jun 18;25:247. doi: 10.1186/s12931-024-02871-6 (PMC11186220; doi:10.1186/s12931-024-02871-6)
Supplement: Supplementary file 1 — Supplementary Material 1 [file 12931_2024_2871_MOESM1_ESM.docx]

SUPPLEMENTAL MATERIAL

**Supplemental table 1**

|  | **OSA (N 16)** |  |  |  | **CSA (N 9)** |  |  |  | **SSH (N 4)** |  |  |  | **No-SDB (N 14)** | |  |
| --- | --- | --- | --- | --- | --- | --- | --- | --- | --- | --- | --- | --- | --- | --- | --- |
|  | **Baseline** | **1-year** | **p.value** |  | **Baseline** | **1-year** | **p.value** |  | **Baseline** | **1-year** | **p.value** |  | **Baseline** | **1-year** | **p.value** |
| EQ5D5L VAS, Median [Q1;Q3] | 62.5 [50;80] | 70 [65;80] | 0.067 |  | 70 [50;70] | 65 [50;70] | 0.810 |  | 65 [50;77.5] | 65 [50;77.5] | 0.850 |  | 75 [68.5;82.5] | 70 [56.25;82.5] | 0.285 |
| KBILD score, Median [Q1;Q3] | 62.2 [53.22;73.7] | 62.3 [54.32;73.7] | 0.433 |  | 61 [53.5;75.2] | 61 [51;75.2] | 0.834 |  | 62.05 [57.47;58.95] | 49.75 [48.78;58.95] | 0.125 |  | 58.1 [53.38;69.08] | 54.75 [51.77;69.08] | 0.67 |
| GERDQ, Median [Q1;Q3] | 6 [4.75;7] | 6 [6;7] | 0.370 |  | 6 [6;6] | 6 [6;6] | 0.833 |  | 6 [6;7] | 4.5 [2.5;7] | 0.371 |  | 6 [5.25;8.75] | 7.5 [6;8.75] | 0.247 |
| GAD7, Median [Q1;Q3] | 1 [0;5] | 1 [0;5] | 0.156 |  | 4 [1;12] | 3 [2;12] | 0.400 |  | 0 [0;2] | 0 [0;2] | 1.000 |  | 3.5 [0.25;10] | 5 [2;10] | 0.305 |
| BECK, Median [Q1;Q3] | 6.5 [2;11.5] | 7.5 [1.75;11.5] | 0.172 |  | 7 [5;19] | 10 [3;19] | 0.360 |  | 5.5 [3;12.75] | 11.5 [8.25;12.75] | 0.174 |  | 7.5 [2.5;16] | 9 [4.25;16] | 0.482 |
| FOSQ, Median [Q1;Q3] | 19.7 [18.78;20] | 19.83 [18.94;20] | 0.255 |  | 19.4 [18.5;20] | 19.67 [13.67;20] | 0.441 |  | 20 [18.23;19.58] | 18.97 [17.08;19.58] | 0.181 |  | 18.55 [17.55;20] | 19.48 [17.33;20] | 0.683 |
| ESS, Median [Q1;Q3] | 8.5 [4.75;8] | 5.5 [4;8] | 0.01 |  | 8 [4;8] | 6 [3;8] | 0.181 |  | 4.5 [3;11.5] | 7.5 [3.5;11.5] | 0.586 |  | 9 [5.5;8.75] | 6 [3.5;8.75] | 0.342 |

OSA: obstructive sleep apnea; CSA: central sleep apnea; SSH: sleep sustained hypoxemia; SBD: sleep-disordered breathing; ESS: Epworth sleepiness scale

**Supplemental table 2. Sleep treatment side effects**

|  | **1 month** | **1 year** |
| --- | --- | --- |
|  | *N=26* | *N=29* |
| Nasal congestion, N (%): | 1 (3.85%) | 3 (10.3%) |
| Air leak, N (%): | 1 (3.85%) | 3 (10.3%) |
| Skin problems, N (%): | 0 (0.00%) | 1 (3.45%) |
| Dry mouth, N (%): | 6 (23.1%) | 8 (27.6%) |
| Noise, N (%): | 2 (7.69%) | 2 (6.90%) |
| Conjunctivitis, N (%): | 0 (0.00%) | 3 (10.3%) |
| Headache, N (%): | 0 (0.00%) | 1 (3.45%) |
| Insomnia, N (%): | 0 (0.00%) | 2 (6.90%) |
| Heat, N (%): | 1 (3.85%) | 3 (10.3%) |
| Abdominothoracic discomfort, N (%): | 0 (0.00%) | 1 (3.45%) |
|  |  |  |

**Supplemental table 3. Patient satisfaction**

|  | **[ALL]** | **OSA** | **CSA** | **SSH** | **No SDB** |
| --- | --- | --- | --- | --- | --- |
|  | *N=43* | *N=16* | *N=9* | *N=4* | *N=14* |
| **Are you satisfied with the sleep follow-up conducted? N (%):** |  |  |  |  |  |
| No | 0 (0.00%) | 0 (0.00%) | 0 (0.00%) | 0 (0.00%) | 0 (0.00%) |
| Partially | 2 (4.65%) | 0 (0.00%) | 1 (11.1%) | 0 (0.00%) | 1 (7.14%) |
| Yes | 41 (95.3%) | 16 (100%) | 8 (88.9%) | 4 (100%) | 13 (92.9%) |
| **Has access to healthcare professionals and information been easy? N (%):** |  |  |  |  |  |
| No | 0 (0.00%) | 0 (0.00%) | 0 (0.00%) | 0 (0.00%) | 0 (0.00%) |
| Partially | 2 (4.65%) | 1 (6.25%) | 1 (11.1%) | 0 (0.00%) | 0 (0.00%) |
| Yes | 41 (95.3%) | 15 (93.8%) | 8 (88.9%) | 4 (100%) | 14 (100%) |
| **Did you feel secure regarding the confidentiality of your data? N (%):** |  |  |  |  |  |
| No | 0 (0.00%) | 0 (0.00%) | 0 (0.00%) | 0 (0.00%) | 0 (0.00%) |
| Partially | 0 (0.00%) | 0 (0.00%) | 0 (0.00%) | 0 (0.00%) | 0 (0.00%) |
| Yes | 43 (100%) | 16 (100%) | 9 (100%) | 4 (100%) | 14 (100%) |
| Have you been treated respectfully and kindly? N (%): |  |  |  |  |  |
| No | 0 (0.00%) | 0 (0.00%) | 0 (0.00%) | 0 (0.00%) | 0 (0.00%) |
| Partially | 0 (0.00%) | 0 (0.00%) | 0 (0.00%) | 0 (0.00%) | 0 (0.00%) |
| Yes | 43 (100%) | 16 (100%) | 9 (100%) | 4 (100%) | 14 (100%) |
| Did you feel safe during the sleep study?, N (%): |  |  |  |  |  |
| No | 0 (0.00%) | 0 (0.00%) | 0 (0.00%) | 0 (0.00%) | 0 (0.00%) |
| Partially | 1 (2.33%) | 0 (0.00%) | 0 (0.00%) | 0 (0.00%) | 1 (7.14%) |
| Yes | 42 (97.7%) | 16 (100%) | 9 (100%) | 4 (100%) | 13 (92.9%) |
| Would you consider doing the sleep study again or would you recommend it to others?, N (%): |  |  |  |  |  |
| No | 0 (0.00%) | 0 (0.00%) | 0 (0.00%) | 0 (0.00%) | 0 (0.00%) |
| Partially | 2 (4.65%) | 1 (6.25%) | 0 (0.00%) | 0 (0.00%) | 1 (7.14%) |
| Yes | 41 (95.3%) | 15 (93.8%) | 9 (100%) | 4 (100%) | 13 (92.9%) |
| Satisfaction level (minimum 0-10 maximum) |  |  |  |  |  |
| Median [Q1;Q3] | 10.0 [9.00;10.0] | 9.50 [9.00;10.0] | 9.00 [9.00;10.0] | 10.0 [9.50;10.0] | 10.0 [9.25;10.0] |
|  |  |  |  |  |  |

OSA: obstructive sleep apnea; CSA: central sleep apnea; SSH: sleep sustained hypoxemia; SBD: sleep-disordered breathing
